# Adjunctive Systemic Corticosteroids for Hospitalized Community-Acquired Pneumonia: Systematic Review and Meta-Analysis 2015 Update

**DOI:** 10.1038/srep14061

**Published:** 2015-09-16

**Authors:** Nobuyuki Horita, Tatsuya Otsuka, Shusaku Haranaga, Ho Namkoong, Makoto Miki, Naoyuki Miyashita, Futoshi Higa, Hiroshi Takahashi, Masahiro Yoshida, Shigeru Kohno, Takeshi Kaneko

**Affiliations:** 1 Department of Pulmonology, Yokohama City University Graduate School of Medicine, Yokohama, Japan; 2 Department of Pulmonology, Tohoku Rosai Hospital, Sendai, Japan; 3 Department of Infectious Diseases, Respiratory and Digestive Medicine, Faculty of Medicine, University of the Ryukyus, Okinawa, Japan; 4 Division of Pulmonary Medicine, Department of Medicine, Keio University School of Medicine, Tokyo, Japan; 5 Department of Respiratory Medicine, Japanese Red Cross Sendai Hospital, Sendai Japan; 6 Department of Internal Medicine I, Kawasaki Medical School, Okayama, Japan; 7 National Hospital Organization Okinawa National Hospital, Okinawa, Japan; 8 Department of Respiratory Medicine, Saka General Hospital, Miyagi, Japan; 9 Department of Hemodialysis and Surgery, Chemotheraphy Research Institute, International University of Health and Welfare, Ichikawa, Japan; 10 Department of Molecular Microbiology and Immunology, Nagasaki University Graduate School of Biomedical Sciences, Nagasaki, Japan

## Abstract

Previous randomized controlled trials (RCTs) and meta-analyses evaluated the efficacy and safety of adjunctive corticosteroids for community-acquired pneumonia (CAP). However, the results from them had large discrepancies. The eligibility criteria for the current meta-analysis were original RCTs written in English as a full article that evaluated adjunctive systemic corticosteroids adding on antibiotic therapy targeting typical and/or atypical pathogen for treating hospitalized human CAP cases. Four investigators independently searched for eligible articles through PubMed, Embase, and Cochrane databases. Random model was used. The heterogeneity among original studies and subgroups was evaluated with the I^2^ statistics. Of 54 articles that met the preliminary criteria, we found 10 eligible RCTs comprising 1780 cases. Our analyses revealed following pooled values by corticosteroids. OR for all-cause death: 0.80 (95% confidence interval (95% CI) 0.53–1.21) from all studies; 0.41 (95% CI 0.19–0.90) from severe-case subgroup; 0.21 (95% CI 0.0–0.74) from intensive care unit (ICU) subgroup. Length of ICU stay: −1.30 days (95% CI (−3.04)−0.44). Length of hospital stay: −0.98 days (95% CI (−1.26)–(−0.71)). Length to clinical stability: −1.16 days (95% CI (−1.73)–(−0.58)). Serious complications do not seem to largely increase by steroids. In conclusion, adjunctive systemic corticosteroids for hospitalized patients with CAP seems preferred strategies.

Respiratory infection including pneumonia is the third-leading cause of death worldwide^1^,^2^,^3^. A majority of patients with pneumonia presents with community-acquired pneumonia (CAP). Despite recent advances in diagnostic methods and antibiotic treatment, the overall mortality rate of CAP remains approximately 5–20%. Although it had been considered that corticosteroids may exacerbate the control of infectious disease, the recent consensus is that adjunctive corticosteroids added to antibiotic treatment is a reasonable strategy when treating patients with critical infectious diseases namely bacterial meningitis, sepsis and septic shock^4^,^5^. The therapeutic efficacy of corticosteroids may be based on down regulation of excessive inflammation via controlling cytokines. Similarly, corticosteroids attenuate the action of many cytokines involved in the inflammatory response associated with CAP^6^. Thus, the use of adjunctive corticosteroids is an appealing option for CAP treatment and has been evaluated in many randomized controlled trials (RCTs) for decades^7^,^8^,^9^,^10^,^11^,^12^,^13^,^14^,^15^,^16^. However, the results from previously reported RCTs that evaluated the impact of systemic corticosteroids for CAP had large discrepancies with each other. In addition, meta-analyses of these RCTs did not reach complete agreement^12^,^13^,^14^,^15^,^16^,^17^,^18^,^19^,^20^,^21^,^22^ (Table 1). Furthermore, a middle-scale and a large scale RCTs published in 2015 were not included in these published meta-analysis^15^,^16^. Moreover, some recent studies were focused on the time to clinical stability^11^,^15^,^16^, however, none of the previous meta-analyses evaluated the time to clinical stability. Therefore, we believe an updated meta-analysis is required. The aim of this study was to evaluate the efficacy and safety of adjunctive corticosteroids for patients with CAP.

## Methods

### Study search

Institutional review board approval and patient consent were not required because of the review nature of this study.

Four investigators independently searched for eligible articles as of March 2015 through PubMed, Embase, and Cochrane databases. One reviewer used the following search formula for PubMed: “(community acquired pneumonia) AND (RCT OR randomized) AND (steroid OR corticosteroid OR cortisol OR methylprednisolone OR prednisolone OR dexamethasone OR hydrocortisone)”.

The eligibility criteria for the current meta-analysis were original RCTs written in English as a full article that evaluated adjunctive systemic corticosteroids adding on antibiotic therapy targeting typical and/or atypical pathogen for treating hospitalized human CAP cases. Any types, any doses, and any durations of systemic corticosteroids were allowed. Articles focusing on an immunocompromised host, inhaled corticosteroid, nosocomial pneumonia, child cases, or specified pathogens were excluded. Included articles had to evaluate at least one of the outcomes mentioned below.

### Outcomes

Mortality that can be expressed as an odds ratio (OR) was evaluated. If two or more types of mortality were descripted, the mortality of longer duration was selected. For example, 90-day mortality was preferred followed by 30-day, in-hospital, intensive care unit (ICU) mortality, in that order.

Length of ICU stay, length of hospital stay, and length to clinical stability in the form of a hazard ratio (HR) or mean difference were also meta-analyzed.

### Statistics

If both an intention-to-treat analysis and per-protocol-analysis were conducted, we adopted the result from the intention-to-treat analysis. Throughout the meta-analyses random model, not fixed model, was used^23^. If necessary, mean and standard deviation (SD) of length of ICU stay, length of hospital stay, and length to clinical stability were estimated from median, range, or interquartile range of them^23^,^24^.

The heterogeneity among original studies and subgroups was evaluated with the I^2^ statistics whereby I^2 ^= 0% indicates no heterogeneity, 0% < I^2 ^< 25% indicates the least heterogeneity, 25% ≤ I^2 ^< 50% indicates mild heterogeneity, 50% ≤ I^2 ^< 75% indicates moderate heterogeneity, and 75% ≤ I^2^ indicates strong heterogeneity^25^.

## Results

### Study search

**Figure 1:**
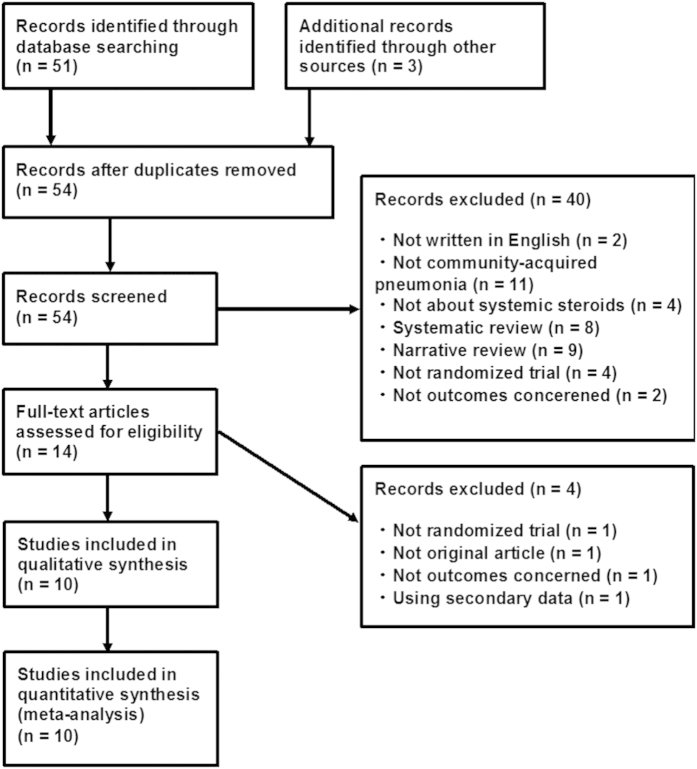
PRISMA diagram.

Of 54 articles that met the preliminary criteria, we found 10 eligible RCTs^7^,^8^,^9^,^10^,^11^,^12^,^13^,^14^,^15^,^16^ (Fig. 1).

These 10 RCTs include four single-facility trials and six multiple-facility trials. Concerning blinding, seven were blinded for both physicians and patients, one was blinded for only patients, and two were not blinded. Prednisolone (PSL), hydrocortisone (HC), methylprednisolone (mPSL), and dexamethasone (DEX) were administered for four, three, two, and one trial, respectively. One study used HC bolus only, while the others administered corticosteroids for three to nine days. The numbers of cases included in each study ranged from 30 to 785 with a median of 100. The average ages of patients in each study were early 60 s for seven studies. In each study, 48–73% of cases were men (Table 2). Each study had a low or unclear risk of bias (Table 3).

### Mortality

All included studies, except for one reported mortality. A report from Mikami did not report data concerning mortality^10^. Moreover, no response to our e-mail inquiry was obtained from the author. Thus, data concerning mortality in this report was derived from a systematic review by Salluh *et al.*^17^.

**Figure 2:**
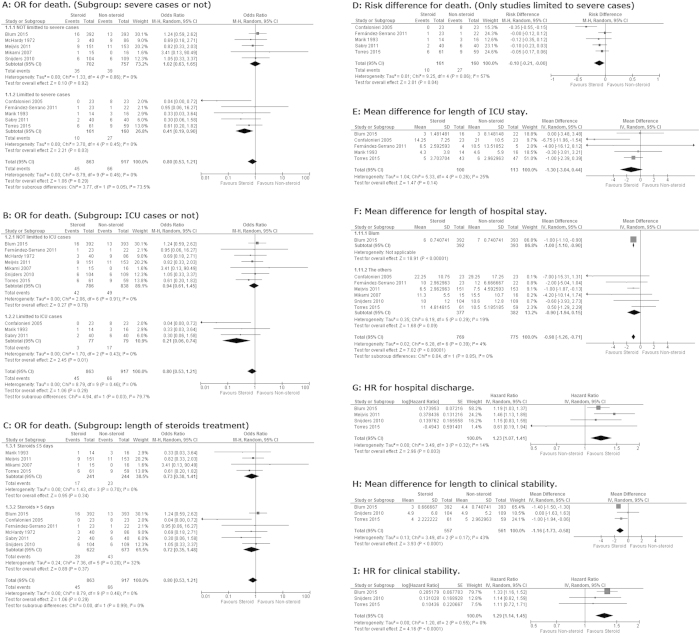
Forrest plots. OR: odds ratio. HR: hazard ratio. 95% CI: 95% confidence interval. M-H: Mantel-Haenzel. ICU: intensive care unit.

Pooled OR for all-cause death by adjunctive corticosteroids estimated from the results of 10 RCTs was 0.80 (95% confidence interval (95% CI) 0.53–1.21, *P *= 0.29. I^2 ^= 0%, *P* for heterogeneity = 0.46) (Fig. 2A).

Because previous meta-analyses suggested that systemic corticosteroids improve the mortality only for severe CAP^17^,^20^,^21^, we performed a subgroup sensitive analysis dividing studies into those with severe case only and those not limited to severe cases. Previous meta-analyses used different criteria of “severe pneumonia^17^,^20^,^21^”. Here, we selected studies that clearly indicated “severe CAP” in the title and/or abstract and studies limited to ICU cases. We observed a strong subgroup difference between the results from five trials not limited to severe cases and those from five trials limited to severe cases (I^2 ^= 73.5%, *P* for subgroup heterogeneity = 0.05) (Fig. 2A). Five RCTs limited to severe cases yielded a pooled OR of 0.41 (95% CI 0.19–0.90, *P *= 0.03. I^2 ^= 0%, *P* for heterogeneity = 0.45) (Fig. 2A). In addition, we conducted another subgroup analysis dividing studies into those limited to ICU cases and those not limited to ICU cases. Again, we observed a strong subgroup difference (I^2 ^= 79.7%, *P* for subgroup heterogeneity = 0.03). Three RCTs limited to ICU cases yielded a pooled OR of 0.21 (95% CI 0.06–0.74, *P *= 0.01. I^2 ^= 0%, *P* for heterogeneity = 0.43) (Fig. 2B).

Since Nie *et al.* reported that CS > 6 d was more beneficial than CS ≤ 5 d^20^, we performed a sensitivity analysis. However, we could not find a significant subgroup OR difference between prolonged and short corticosteroids treatment for all-cause mortality. (I^2 ^= 0%, *P* for subgroup heterogeneity = 0.99) (Fig. 2C).

Using data from the five trials limited to severe cases, the pooled risk difference for mortality was −0.10 (95% CI –(0.21)–(−0.00), *P *= 0.04). This pooled difference of −0.10 was equivalent to a number needed to treat of 10 (Fig. 2D).

### Length of ICU stay

A random-model meta-analysis using data from five RCTs indicated that length of ICU stay slightly favored the steroid arm but was not significantly different between the two arms with a mean difference of −1.30 days (95%CI (−3.04)–0.44, *P *= 0.14. I^2 ^= 25%, *P* for heterogeneity = 0.26) (Fig. 2E).

No study reported HR for length of ICU stay.

### Length of hospital stay

Seven studies reported difference of length of hospital stay in days. Based on these data, the length of hospital stay was shorter in the steroid arm with a pooled mean difference of −0.98 (95% CI (−1.26)–(−0.71), *P *< 0.001. I^2 ^= 4%, *P* for heterogeneity = 0.39). We performed a sensitivity analysis comparing the result from a study by Blum *et al.* and those from the others because the study by Blum *et al.* had very large weight of 86.8%. However, there was no significant subgroup heterogeneity between them (I^2 ^= 0%, *P* for subgroup heterogeneity = 0.85) (Fig. 2F).

When evaluated using HR for hospital discharge, the length in hospital stay was also shorter in the steroid arm with a pooled HR of 1.23 (95% CI 1.07–1.41, *P *= 0.003. I^2 ^= 14%, *P* for heterogeneity = 0.32) (Fig. 2G).

### Length to clinical stability

Length to clinical stability was evaluated in three recent studies. They yielded pooled difference of −1.16 days (95% CI (−1.73)–(−0.58), *P *< 0.001. I^2 ^= 43%, *P* for heterogeneity = 0.17) (Fig. 2H).

The same three studies yielded a pooled HR of 1.29 (95% CI 1.14–1.45, *P *< 0.001. I^2 ^= 0%, *P* for heterogeneity = 0.55) (Fig. 2I).

### Adverse effects

In this systematic review, we noticed that some reports described adverse effects. However, these data were not suitable for qualitative meta-analysis due to discrepancy of descriptive method.

Confalonieri *et al.* reported that major complication (steroid: 6/23 vs. placebo 18/23, *P *< 0.001) and delayed septic shock (steroid: 0/23 vs. placebo: 10/23, *P *< 0.001) were less frequently observed in the steroid arm^9^. Mikami *et al.* reported no significant difference in deterioration of glucose intolerance, electrolyte abnormalities, nor other adverse effects was observed^10^. Snijders *et al.* revealed that the overall adverse effect was not different in two arms (steroid: 63/104 vs. placebo: 72/109, *P *= 0.41)^11^. Meijvis *et al.* reported that hyperglycemia was more common in the steroid arm (steroid: 67/151 vs. placebo: 35/153, *P *< 0.001) but that the requirement for glucose-lowering treatment and superinfection were not significantly different between arms^13^. Blum at al. wrote that overall adverse events were frequently observed in the steroid arm (steroid: 96/392 vs. placebo: 61/393, *P *< 0.001), and the difference was mainly attributed to the increase of hyperglycemia requiring new insulin (steroid: 76/392 vs. placebo: 43/393, *P *= 0.001)^15^. Torres *et al.* compared the incidence of six adverse effects including hyperglycemia but none differed significantly^16^.

In general, although patients in the steroid arm might have a higher risk for overall adverse events and hyperglycemia, serious complications do not seem to largely increase due to use of systemic steroids.

## Discussion

This updated systematic review and meta-analysis had the largest sample size ever (Table 1). The key findings from this analysis are as follows: (i) corticosteroids shortens length of hospital stay for CAP, (ii) corticosteroids shortens length to clinical stability for CAP, and (iii) corticosteroids lowers mortality for severe CAP. Additionally, we revealed that the incidence of major complications was not greatly increased by systemic steroids, though quantitative synthesis was not feasible.

Inflammation is part of the complex biological response to harmful stimuli such as pathogens and damaged cells. This response is essential to control CAP. However, fulminant inflammation often results in organ failure. The rationale of using adjunctive systemic corticosteroids for CAP is to down-regulate excessive inflammation. Corticosteroids are very potent and by far the most frequently used type of immunosuppressant. The mechanism of corticosteroids is not fully clear; however, it is believed that corticosteroids switch off genes that encode pro-inflammatory cytokines and switch on genes that encode anti-inflammatory cytokines^6^. Based on our analysis, corticosteroids decreased mortality for severe/ICU CAP only. In this subgroup, merit of controlling systemic inflammation may overweight demerit of deteriorating innate body antibacterial action due to immunosuppressant.

Except for the OR for death, frequently used outcomes that we also evaluated in this study need be interpreted carefully. The length of hospital stay and ICU stay are often affected by non-pharmacological reasons, such as availability of ICU and non-ICU beds in a hospital. In this view, the time to clinical stability can evaluate more physiological aspects of a patient. However, some items included in the definition of clinical stability are known to be relieved by the administration of the systemic corticosteroids without direct relationship with the mortality^26^. For example, strong antipyretic effect of the corticosteroids obviously shortens the time to clinical stability. However, it is not clear whether the antipyretic effect can improve mortality and other outcomes. But then again, corticosteroids favored results for these uncertain outcomes, together with the decreased OR for death, made our results plausible.

The best corticosteroids regimen, for CAP is not clear. Nie *et al.* found that prolonged use of corticosteroids (>5 days) was associated with a greater benefit compared with short course corticosteroids (≤5 days)^20^. However, this was not replicated in the current analysis. The length of the corticosteroids therapy used in the included trials was five days on average with a median of seven days (Table 2). Thus, a 5–7 days treatment course seems reasonable for now. The best dosage of corticosteroids is also unclear. Most studies used HC 200–300 mg/day, PSL/mPSL 20–50 mg/day, or mPSL 1mg/kg/day (Table 2). Thus, a so-called low- or middle-dose of corticosteroids seems an acceptable choice in practice. Corticosteroids in these doses are also chosen for treatment of other common form of critical systematic inflammatory disease such as acute exacerbation of chronic obstructive pulmonary disease^27^, acute respiratory distress syndrome^28^, and septic shock^29^.

Some studies that were not included in this study have also revealed the efficacy of systematic corticosteroids for CAP. Garcia-Vidal *et al.* conducted a retrospective study with 308 severe CAP patients, which concluded that risk of death was reduced with adjusted OR of 0.287^30^. A cohort study in 2013 by Nagy *et al.* reported that the 5-day methylprednisolone therapy with imipenem was found to be effective in children with severe CAP^31^. Adjunctive systemic corticosteroids may be effective for CAP patients of any age.

Limitations of this study should be mentioned. First, we could not meta-analyze the data for adverse effects. Second, although four studies that evaluated the length of ICU stay constantly indicated the tendency of a steroid-favored outcome, these results did not reach statistical significance probably due to lack of power. Third, we could not infer the impact of corticosteroids for outpatients with CAP. Forth, the best corticosteroids regimen could not be clarified. Lastly, our analysis could not directly explain why the adjunctive corticosteroids have different impact for mild and severe CAP.

In conclusion, although further RCTs focusing on severe CAP are anticipated, adjunctive systemic corticosteroids for hospitalized patients with CAP seem preferred strategies because this treatment shortens the length of hospital stay and the length to clinical stability without increasing severe adverse effect. In addition, adjunctive systemic corticosteroids lowers the mortality of severe CAP cases. Prolonged use of corticosteroids (>5 days) was not more effective than a short course of corticosteroids (≤5 days). The best corticosteroids regimen is not clear, however, a middle dose of corticosteroids, for example PSL/mPSL 40 mg/day to 1 mg/kg/day for 5–7 days seems a reasonable choice.

## Additional Information

**How to cite this article**: Horita, N. *et al.* Adjunctive Systemic Corticosteroids for Hospitalized Community-Acquired Pneumonia: Systematic Review and Meta-Analysis 2015 Update. *Sci. Rep.* **5**, 14061; doi: 10.1038/srep14061 (2015).

## Figures and Tables

**Table 1:** Systematic reviews and meta-analyses evaluating adjunctive corticosteroids for community-acquired pneumonia.

Author, year	Severity and type of pneumonia	Number of studies	number of cases	Conclusion
Salluh, 2008^17^	Severe CAP	3 RCTs and 1 cohort study	415	Not recommending CS for severe CAP.
Siempos, 2008^18^	CAP with any severity (only studies for severe CAP were found)	4 RCTs	189	Limited data suggested that CS lowers mortality and shortens length of hospital stay for severe CAP.
Chen, 2011^19^	Any type of pneumonia. including nosocomial and child pneumonia	6 RCTs (2 for child, 1 for inhaled CS.	437	CS are generally beneficial for pneumonia. Evidence is lacking to make recommendation.
Nie, 2012^20^	CAP with any severity.	9 RCTs	1001	CS is not recommended for CAP in general. However, it is possible that CS lower mortality from severe CAP and that CS > 6 d may be more beneficial than CS ≤ 5 d.
Cheng, 2013^21^	Severe CAP	4 RCTs	264	Limited evidence suggests that CS lowers mortality from severe CAP.
Shafiq, 2013^22^	CAP requiring admission.	8 RCTs	1119	CS shortens length of hospital stay. CS does not lower mortality.
Current study	CAP requiring admission.	10 RCTs	1780	CS shortens length of hospital stay for CAP. CS shortens length to clinical stability for CAP. CS lowers mortality for CAP in intensive care unit. CS > 6 d is not more beneficial than CS ≤ 5 d.

[ ]: reference number.

RCT: randomized controlled trial. CS: corticosteroids. CAP: community-acquired pneumonia.

**Table 2:** Characteristics of included randomized controlled trials.

Study	Origin of report	blinding	facility	Age criteria	Severity criteria	Steroid regimen	Antibiotics	Number of patients Steroid + Non-steroid = Total	Average age	Men%
McHardy 1972^7^	Australia	Not-blinded	Single	12-	not at risk of dying within 24 hour	PSL 20 mg/day for 7 d	Ampicillin 1 or 2 g/day	40 + 86 = 126	61	48%
Marik 1993^8^	USA South Africa	Single-blinded	Multi	18–70	ICU, BTS criteria severe	HC 10 mg/kg bolus	Cefotaxime based regimen	14 + 16 = 30	36	NS
Confalonieri 2005^9^	Italy	Double-blinded	Multi	not specified	ICU, ATS criteria severe	HC 200 mg bolus + 240 mg/day for 7 d	Based on guidelines	23 + 23 = 46	64	70%
Mikami 2007^10^	Japan	Not-blinded	Single	adult	Moderate to severe. Not ventilated at admission	PSL 40 mg/day for 3 d	Ampicillin/Sulbactam and Carbapenem were preferred	15 + 16 = 31	72	74%
Snijders 2010^11^	Netherlands	Double-blinded	Single	18-	NS	PSL 40 mg/day for 7 d	Amoxicillin and Moxifloxacin were preferred	104 + 109 = 213	64	58%
Fernández-Serrano2011^12^	Spain	Double-blinded	Single	18–75	P/F ratio < 300, Not ventilated at admission	mPSL 200 mg bolus + 80 mg/day for 3 days + 40 mg/day for 3 days + 20 mg/ady for 3 days.	Ceftriaxone + Levofloxacin	22 + 23 = 45	64	67%
Meijvis 2011^13^	Netherlands	Double-blinded	Multi	18-	Non-ICU	DEX 5 mg/day for 4 d	Amoxicillin/Clavulanate and Cephalosporin were preferred.	151 + 153 = 304	64	56%
Sabry 2011^14^	Egypt	Double-blinded	Multi	adult	ICU Ventilated	HC 200 mg bolus + 300 mg/day for 7 d	NS	40 + 40 = 80	62	73%
Blum 2015^15^	Swizerland	Double-blinded	Multi	18-	NS	PSL 50 mg/day for 7 d	Based on guidelines	392 + 393 = 785	73	62%
Torres 2015^16^	Spain	Double-blinded	Multi	18-	“ATS criteria severe” or “Pneumonia severity index = V and CRP > 150 mg/L”	mPSL 1 mg/kg/day for 5 d	Ceftriaxon + Levofloxacin or + Azithromycin were preferred	61 + 59 = 120	65	62%

[ ]: reference number.

ICU: intensive care unit. BTS: Brithsh Thoracic Society. ATS: American Thoracic Society. PORT

PSL: prednisolone. mPSL: methylprednisolone. DEX: dexamethasone. HC: hydrocortisone

d: day.

**Table 3:** Risk of bias.

Study Author, year	Randomization Method	Allocation Concealment	Blinding of participants and personnel	Blinding of outcome assessment	Incomplete outcome data	Selective reporting	Other bias	Overall risk
McHardy 1972	Low	Unclear(a)	Unclear(b)	High(c)	Low	Low	Low	**Unclear**
Marik 1993	Low	Unclear(a)	Low	High(c)	Low	Low	Unclear(f)	**Unclear**
Confalonieri 2005	Low	Low	Low	Low	Low	Low	Unclear(f)	**Low**
Mikami 2007	Low	Unclear(a)	Unclear(b)	High(c)	Low	Unclear(e)	Unclear(f)	**Unclear**
Snijders 2010	Low	Low	Low	Low	Low	Low	Low	**Low**
Fernández-S. 2011	Low	Unclear(a)	Low	Low	Unknown(d)	Low	Unclear(f)	**Low**
Meijvis 2011	Low	Low	Low	Low	Low	Low	Low	**Low**
Sabry 2011	Low	Unclear(a)	Low	Low	Low	Low	High(g)	**Unclear**
Blum 2015	Low	Low	Low	Low	Low	Low	Low	**Low**
Torres 2015	Low	Unclear(a)	Low	Low	Low	Low	Low	**Low**

(a) Not centralized randomization.

(b) Open-label study.

(c) Open-label or single-blinded study.

(d) 20% of cases were excluded after randomization and per-protocol analysis was conducted.

(e) Mortality was not reported.

(f) Considering relatively small sample size (n < 50), possible publication bias could not denied.

(g) Insufficient description of methods and results.
